# Phytoplankton-derived polysaccharides and microbial peptidoglycans are key nutrients for deep-sea microbes in the Mariana Trench

**DOI:** 10.1186/s40168-024-01789-x

**Published:** 2024-04-25

**Authors:** Yan-Ru Dang, Qian-Qian Cha, Sha-Sha Liu, Shu-Yan Wang, Ping-Yi Li, Chun-Yang Li, Peng Wang, Xiu-Lan Chen, Ji-Wei Tian, Yu Xin, Yin Chen, Yu-Zhong Zhang, Qi-Long Qin

**Affiliations:** 1 State Key Laboratory of Microbial Technology, Shandong University, Qingdao, China; 2 College of Marine Life Sciences & Frontiers Science Center for Deep Ocean Multispheres and Earth System, Ocean University of China, Qingdao, China; 3 Laboratory for Marine Biology and Biotechnology, National Laboratory for Marine Science and Technology, Qingdao, China; 4 School of Life Sciences, University of Warwick, Coventry, CV4 7AL UK; 5 Marine Biotechnology Research Center, State Key Laboratory of Microbial Technology, Shandong University, Qingdao, China

**Keywords:** Mariana Trench, Biopolymers, Deep-sea microbiome, Hadalpelagic zone, Metatranscriptome, Extracellular enzymes

## Abstract

**Background:**

The deep sea represents the largest marine ecosystem, driving global-scale biogeochemical cycles. Microorganisms are the most abundant biological entities and play a vital role in the cycling of organic matter in such ecosystems. The primary food source for abyssal biota is the sedimentation of particulate organic polymers. However, our knowledge of the specific biopolymers available to deep-sea microbes remains largely incomplete. One crucial rate-limiting step in organic matter cycling is the depolymerization of particulate organic polymers facilitated by extracellular enzymes (EEs). Therefore, the investigation of active EEs and the microbes responsible for their production is a top priority to better understand the key nutrient sources for deep-sea microbes.

**Results:**

In this study, we conducted analyses of extracellular enzymatic activities (EEAs), metagenomics, and metatranscriptomics from seawater samples of 50–9305 m from the Mariana Trench. While a diverse array of microbial groups was identified throughout the water column, only a few exhibited high levels of transcriptional activities. Notably, microbial populations actively transcribing EE genes involved in biopolymer processing in the abyssopelagic (4700 m) and hadopelagic zones (9305 m) were primarily associated with the class Actinobacteria. These microbes actively transcribed genes coding for enzymes such as cutinase, laccase, and xyloglucanase which are capable of degrading phytoplankton polysaccharides as well as GH23 peptidoglycan lyases and M23 peptidases which have the capacity to break down peptidoglycan. Consequently, corresponding enzyme activities including glycosidases, esterase, and peptidases can be detected in the deep ocean. Furthermore, cell-specific EEAs increased at 9305 m compared to 4700 m, indicating extracellular enzymes play a more significant role in nutrient cycling in the deeper regions of the Mariana Trench.

**Conclusions:**

Transcriptomic analyses have shed light on the predominant microbial population actively participating in organic matter cycling in the deep-sea environment of the Mariana Trench. The categories of active EEs suggest that the complex phytoplankton polysaccharides (e.g., cutin, lignin, and hemicellulose) and microbial peptidoglycans serve as the primary nutrient sources available to deep-sea microbes. The high cell-specific EEA observed in the hadal zone underscores the robust polymer-degrading capacities of hadal microbes even in the face of the challenging conditions they encounter in this extreme environment. These findings provide valuable new insights into the sources of nutrition, the key microbes, and the EEs crucial for biopolymer degradation in the deep seawater of the Mariana Trench.

Video Abstract

**Supplementary Information:**

The online version contains supplementary material available at 10.1186/s40168-024-01789-x.

## Background

The organic matter (OM) cycle in the deep sea has a profound impact on global climate and environment, maintaining marine biological productivity and ecological equilibrium in the deep sea. Microbes in the deep sea serve as the foundation of these ecosystems and play a pivotal role in the cycling of OM. The main source of energy and nutrients for deep-sea organisms is the primary production originating at the surface of seawater [1]. As it sinks through the mesopelagic zone, its composition becomes harder to characterize by standard chromatographic techniques [2]. The sinking particulate organic matter (POM), primarily comprising proteinaceous materials and polysaccharides derived from phytoplankton and bacteria, can further aggregate into exopolymer particles [3, 4]. Moreover, microbial activities may introduce new cell walls or other biopolymers that are not easily characterized [2]. Due to the low concentration and high complexity of these biopolymers, it is still unclear which kind of biopolymers could be utilized by microbes when they settle into the deep ocean. The critical bottleneck in the utilization of biopolymers, such as proteins and polysaccharides, is the depolymerization process facilitated by extracellular enzymes (EEs) [5, 6]. Extracellular enzymatic activities (EEAs) dictate which types of OM can be degraded and at what rates [7]. Therefore, measuring EEA is essential for gaining a fundamental understanding of the biogeochemical cycling of OM in the ocean [8]. Additionally, changes in microbial EE gene abundance closely correlate with the corresponding enzymatic activity [9, 10]. Thus, microbial EEA serves as an effective indicator of microbial-mediated biopolymer decomposition, providing a reliable estimate of the activity of particular enzymes [9]. However, a comprehensive investigation into the categories and activities of the EEs, as well as the active microbial groups and genes involved in OM processing throughout the water column, especially in the hadalpelagic zone has yet to be systematically conducted [11–16]. The Mariana Trench, reaching a depth of ~ 11,000 m at the Challenger Deep, represents the deepest places on the Earth. Within this extraordinary geographical setting, unique microbial communities thrive, making it an ideal site for investigating vertical variations in the active microbial groups engaged in OM processing throughout the water column, particularly in the hadalpelagic zone.

Microbes mediate a large portion of the energy and matter flow in deep-sea ecosystems. Recent studies using 16S rRNA gene amplicons and metagenomic sequencing have revealed that Proteobacteria were dominant in the hadalpelagic zone of the Mariana Trench and deep-sea microbes had miscellaneous functions, such as hydrocarbon degradation and dimethylsulfoniopropionate production [12, 13]. Metabolic reconstruction of metagenome-assembled genomes (MAGs) highlighted hadopelagic seawater Thaumarchaeota and Nitrospirae driving nitrification, Rhodospirillales facilitating sulfur oxidation, and hadopelagic sediment Chloroflexi harboring pathways for recalcitrant OM degradation [17, 18]. Some deep-sea enriched microbes also had the ability to degrade organic biopolymers, such as alginate, cellulose, pectin, and xylan [19–23].

These studies showed that deep-sea microbes had diverse metabolic potentials and played important roles in the OM cycle. However, the active microbiome involved in OM cycling and associated activities of EE have not been extensively studied. Here, we employ a combination of metagenomic/metatranscriptomic sequencing and EEA measurements to explore the active genes and microbial groups involved in the processing of biopolymers, providing a first insight into the available sources of nutrition and the key enzymes of active microbiome involved in deep-sea OM cycle in the Mariana Trench.

## Results and discussion

### DNA-based and RNA-based taxonomic profiling of the microbial community composition throughout the water column

To determine the vertical distribution pattern and activity of microbial communities throughout the Mariana Trench Challenger Deep, metagenomic (DNA-based) and metatranscriptomic (RNA-based) sequencing and analyses were performed on seawater microbial communities collected from water depths of 50 m, 1045 m, 1743 m, 4700 m, and 9305 m (Table S1 and S2).

**Fig. 1:**
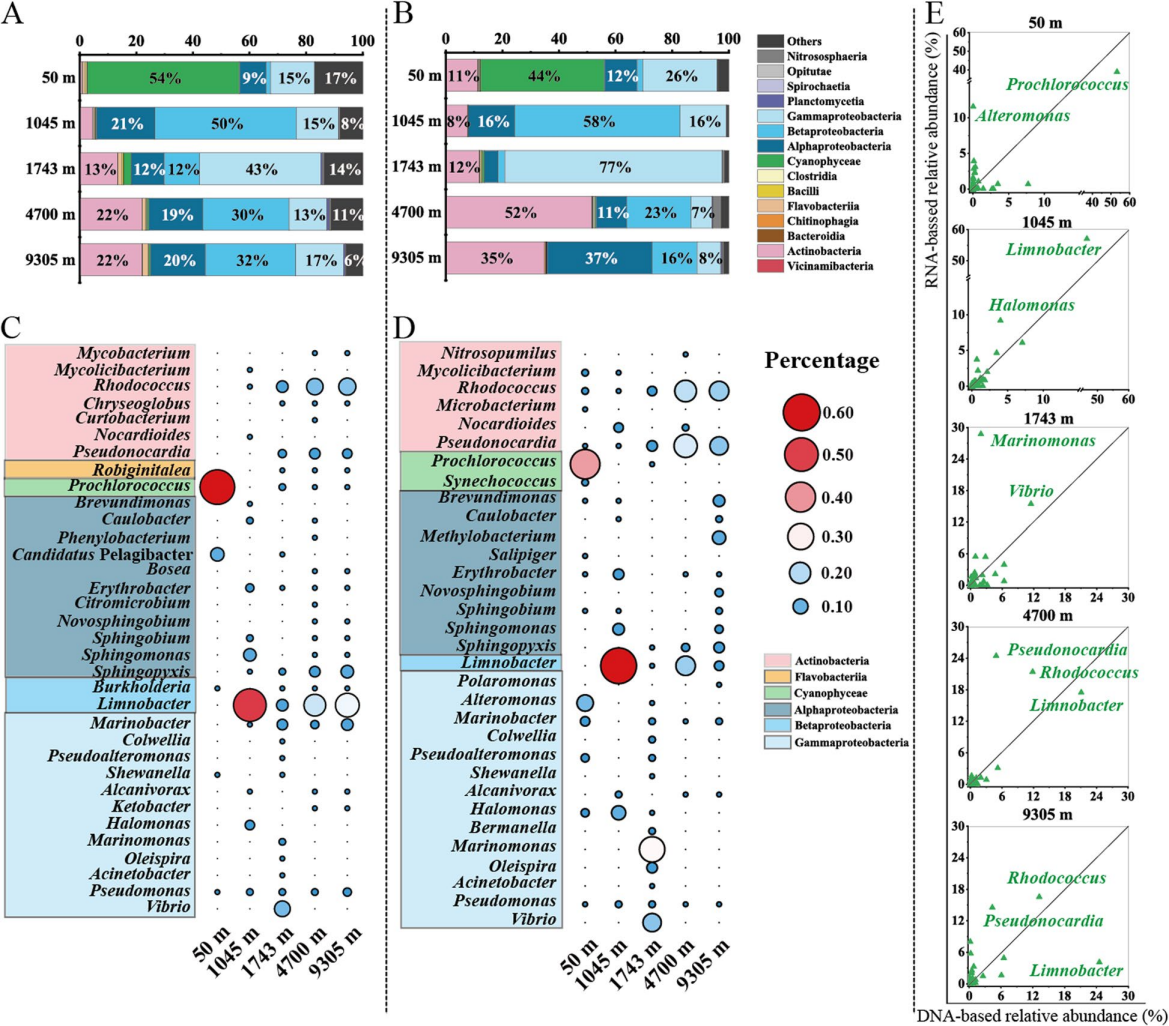
Taxonomic profiling of the microbial community composition throughout the water column. Microbial community profiling at the class level based on the metagenomic (**A**) and metatranscriptomic (**B**) data. Dominant microbial groups at genus level based on the metagenomic (**C**) and metatranscriptomic (**D**) data. **E** Correlation between DNA-based and RNA-based relative abundance at the genus level. Sample names are defined by sampling depth. Dot size in the panel **C** and **D** is proportional to the relative abundance of microbial groups. The genera shadowed in colorful boxes are from the same class group

The taxonomic analysis of metagenomic reads, gene sets and 16S rRNA gene tags in metagenomic reads (16S miTags) showed that Proteobacteria, particularly α-Proteobacteria, β-Proteobacteria, and γ-Proteobacteria, were the predominant microbial lineages across all water depths, except for the epipelagic zone (50 m) where Cyanophyceae was the most abundant group (Fig. 1A, Figure S1A and S1C). In the mesopelagic zone (1045 m), the class β-Proteobacteria constituted the majority (50%). The proportion of class γ-Proteobacteria increased, reaching 43% in the bathypelagic zone (1743 m). However, a high abundance of classes β-Proteobacteria (30%, 32%), α-Proteobacteria (19%, 20%), and Actinobacteria (22%, 22%) in the abyssopelagic (4700 m) and hadopelagic (9305 m) zone, respectively, were detected. The results of 16S miTags analysis showed that the relative abundance of γ-Proteobacteria increased at 9305 m (Figure S1C), which may be caused by different analysis methods. Nevertheless, the relatively high abundances of β-Proteobacteria and Actinobacteria in the hadopelagic zone were not observed in this area [12, 15].

Taxonomic classification of the active microbial communities was based on the non-rRNA reads and transcripts obtained from metatranscriptomic datasets (Fig. 1B and Figure S1B). Taxonomic profiling of the metatranscriptomic reads (RNA-based) revealed a distinct dominant population at each depth. For instance, at 50 m, the class Cyanophyceae constituted 44% of the community, while at 1045 m, β-Proteobacteria dominated, accounting for 58% of the community. At 1743 m γ-Proteobacteria were the most abundant group (77%), whereas Actinobacteria (52%) were the most abundant group at 4700 m and α-Proteobacteria (37%) and Actinobacteria (35%) dominated at 9305 m. Previous studies suggested that γ-Proteobacteria were the main microbial group in the deep sea of the Mariana Trench [16]. However, the RNA-based taxonomic profiling in this study revealed that Actinobacteria, α-Proteobacteria, and β-Proteobacteria groups had higher transcript abundances than the γ-Proteobacteria group in the abyssopelagic and hadalpelagic zones. These findings demonstrate that the active microbial communities vary with water depth, and, in the abyssopelagic and hadopelagic zones, the Actinobacteria and α-Proteobacteria groups emerge as the major active groups.

At the genus level, the DNA-based taxonomic profiling showed that *Prochlorococcus* (Cyanophyceae) and “*Candidatus* Pelagibacter” (α-Proteobacteria) at 50 m, *Limnobacter* (β-Proteobacteria) and *Sphingomonas* (α-Proteobacteria) at 1045 m, *Vibrio* (γ-Proteobacteria) at 1743 m, *Rhodococcus* (Actinobacteria), *Limnobacter*, *Marinobacter* (γ-Proteobacteria) and *Sphingopyxis* (α-Proteobacteria) at 4700 m and 9305 m had high relative abundance (Fig. 1C). However, in contrast to the DNA-based taxonomic profiling, the RNA-based diversity analysis revealed that “*Candidatus* Pelagibacter” did not have high RNA-based abundance at 50 m, and *Limnobacter* did not either at 9305 m (Fig. 1D). The genera *Pseudonocardia* (Actinobacteria) and *Rhodococcus* at 4700 m and 9305 m, and some genera of the α-Proteobacteria group, such as *Brevundimonas* and *Sphingopyxis*, had high RNA-based abundance at 9305 m. Interestingly, our results indicated a significant presence of β-Proteobacteria in the deep-sea environment, especially at 1045 m, which had not been extensively reported before. The high abundance of β-Proteobacteria was mainly due to the substantial presence of the genus *Limnobacter*, accounting for 93% of the β-Proteobacteria abundance at 1045 m. *Limnobacter* has previously been associated with algal blooms and found to be abundant in copepod gastrointestinal tracts [24–27]. The high abundance of *Limnobacter* at 1045 m may be linked to the periodic settlement of planktonic algae and/or the increased copepods activities during the time of sampling in this study.

Next, we conducted a correlation analysis between DNA-based and RNA-based abundance at the genus level (Fig. 1E). In general, when DNA-based and RNA-based abundance in a sample are similar, the abundance values should be distributed around the 1:1 line. However, most abundance values did not fall along the 1:1 line, with several RNA-based abundances being higher than DNA-based abundances. The summed RNA-based abundance of the top 5 genera accounted for 50–81% of the total abundance value of the classified reads, while summed DNA-based abundance accounted for 23–63% of the total abundance value of the classified reads in all samples (Table S3 and S4). Some genera had low DNA-based but high RNA-based abundance, such as *Altermonas* (γ-Proteobacteria) at 50 m, *Halomonas* (γ-Proteobacteria) at 1045 m, *Marinomonas* (γ-Proteobacteria) at 1743 m, *Pseudonocardia* and *Rhodococcus* at 4700 m and 9305 m. Conversely, a few genera displayed high DNA-based but low RNA-based relative abundance, such as *Marinobacter* and *Limnobacter* at 9305 m. The reduced abundance in the hadalpelagic zone suggests that these strains may have been transported into deep seawater by sinking particles from the upper seawater, but were unable to adapt to the deep-sea environment [28]. The above results implied that the transcriptomic data could provide useful complementary information in revealing the active microbial population in the deep sea (e.g., showing the active class Actinobacteria and α-Proteobacteria in 9305 m seawater) that may not be apparent in metagenomic data. Genera with relatively high transcriptional levels such as *Rhodococcus* and *Pseudonocardia*, may play important ecological functions, such as OM degradation and utilization in the hadal region.

### Active microbial communities involved in biopolymer degradation and utilization

The main source of energy and nutrients for deep-sea biota is the sedimentation of POM from the surface seawater [1, 4]. The depolymerization of biopolymers by EEs is considered the pivotal and rate-limiting step in the OM cycling process [5]. To uncover the active microbes involved in biopolymer degradation and utilization, the abundance of genes encoding extracellular degrading enzymes (carbohydrate-active enzymes and peptidases) and transporters (transporting corresponding enzymatic products) throughout the water column were analyzed. For simplicity, the abundance of a gene in a sample deduced from metagenomic data will be referred to as DNA-based abundance, while that obtained from metatranscriptomic data will be referred to as RNA-based abundance.

**Fig. 2:**
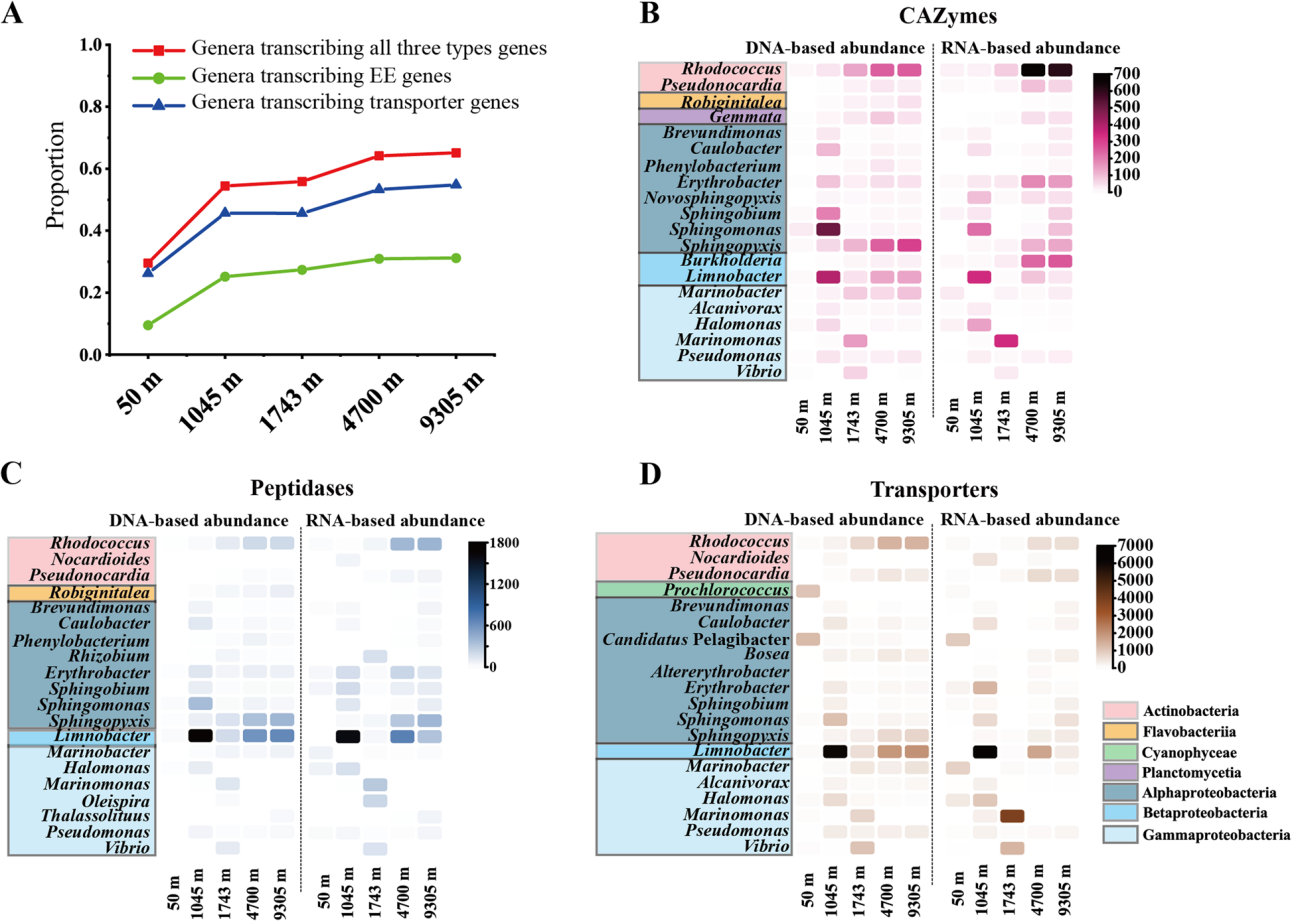
The microbial communities involved in polymer degradation. **A** The proportion of the number of the genera that actively transcribed these three types (CAZyme, peptidase, and transporter) of genes (red boxes), EE genes (green circles), and transporter genes (blue triangles) to the number of the genera that contained these three types of genes, respectively. **B**,** C**, and **D** Dominant genera that contained/transcribed CAZymes (**B**), peptidases (**C**), and transporters (**D**) genes. The DNA-based and RNA-based abundance of these genera were represented by TPM value. The genera shadowed in colorful boxes are from the same class group

There were 420–675 genera that contained genes encoding carbohydrate-active enzymes (CAZymes), peptidases, and transporters, and 124–422 genera that transcribed these three groups of genes throughout the water column (Figure S2). Essentially every genus that was found to be transcribing the gene also encoded for the gene. From metagenomic data, the lowest number of genera containing EE genes was at 50 m, with the highest at 9305 m. Similarly, the lowest number of genera containing transporter genes was observed at 50 m, with the highest at 1743 m. In contrast, from metatranscriptomic data, the number of genera transcribing genes encoding both EE and transporter increased with increasing depth. We noticed that the reads mapping rate was relatively low for the 50 m sample. This would underestimate the proportion of transcribing genes at 50 m to some extent, but would not affect the overall results in deep samples. In general, 30–65% of the genera containing EE and transporter genes were actively transcribing these genes, and this proportion increased with depth (Fig. 2A). The proportion of genera transcribing CAZyme and/or peptidase genes in relation to the number of genera containing these genes increased from 10 to 31% with increasing depth. Similarly, the proportion of genera transcribing transporter genes in relation to the number of genera containing transporter genes increased from 26 to 55% with increasing depth. These findings indicate that the proportion of the genera transcribing the genes involved in degrading and utilizing biopolymers increased with depth.

To further investigate the main genera involved in biopolymer degradation and utilization, the top 20 genera that contained CAZyme, peptidase, and transporter genes with high DNA-based and/or RNA-based abundances were identified (Fig. 2B–D). Out of 20 genera identified, 10 were found to encode and/or transcribe all three groups of genes. However, it is worth noting that the DNA-based and RNA-based abundance of specific genes differed at specific depths. For example, the genes related to CAZyme, peptidase, and transporter from *Limnobacter* had high DNA-based abundance at 1045 m, 4700 m, and 9305 m, but only had high RNA-based abundance at 1045 m and 4700 m. This observation was consistent with the above analysis that the overall activity of *Limnobacter* reduced greatly in 9305 m. The CAZyme, peptidase, and transporter genes from the genus *Marinomonas* had high DNA-based (accounting for up to 52% of the class γ-Proteobacteria) and RNA-based (accounting for up to 84% of the class γ-Proteobacteria) abundance at 1743 m. The CAZyme, peptidase and transporter genes from the genus *Rhodococcus* had high DNA-based (accounting for up to 81% of the class Actinobacteria) and RNA-based (accounting for up to 93% of the class Actinobacteria) abundance at 4700 m and 9305 m, while the CAZyme genes from the genus *Erythrobacter* and *Burkholderia* (β-Proteobacteria) had low DNA-based but high RNA-based abundance at 4700 m and 9305 m. The CAZyme and peptidase genes from the genus *Sphingopyxis* had higher DNA-based abundance (accounting for up to 52% of the class α-Proteobacteria) than RNA-based abundance (accounting for up to 39% of the class α-Proteobacteria) at 4700 m and 9305 m. These analyses showed that active bacteria involved in biopolymer degradation and utilization in the abyssopelagic and hadopelagic zones were mainly from the class Actinobacteria, followed by α-Proteobacteria and β-Proteobacteria. This showed that rare species (with low DNA-based abundances) also can play important ecological functions (with high RNA-based abundances) in the community as reported before [29].

In general, our results indicated that the proportion of active microbial communities involved in biopolymer degradation and utilization increased with depth (Fig. 2A). Previous studies have estimated that direct utilization of low molecular weight (LMW) substances accounted for only ~ 10% of microbial carbon demand in the mesopelagic and bathypelagic waters, based on microbial activity measurements and dissolved organic carbon profiles throughout the water column [30, 31]. LMW substances have a low contribution to supporting heterotrophic microbial metabolism in the deep sea compared to biopolymers, and the quantity and quality of LMW substances decrease with depth [32]. Labile LMW substances were preferentially consumed by microbes, leaving behind complex biopolymers [33]. Therefore, as illustrated in this study, microbial communities in the deep sea must produce EEs to degrade biopolymers and transporters to utilize the enzymatic products in the context of nutritional deficiencies. The dominant microbial population involved in biopolymer degradation and utilization in the deep sea was mainly from the class Actinobacteria, which has been overlooked in the past. Actually, Actinobacteria strains that can degrade biopolymers have been isolated from the Mariana Trench deep water [34] and a high abundance of Actinobacteria encoding CAZymes and peptidases was detected in other deep-sea samples [35], indicating that Actinobacteria is an important group in OM cycling in deep-sea environments.

### Active genes involved in biopolymer degradation and utilization

Having established the dominant and active microbial taxa involved in biopolymer transformation, we next carried out detailed analyses of CAZyme, peptidase, and transporter genes. Generally, the total number of CAZyme, peptidase, and transporter genes inferred from metagenomic data was approximately 2–9 times greater than the number of the actively transcribed genes deduced from metatranscriptomic data at each depth (Figure S3). Only a small proportion of genes had high transcriptional levels at a specific depth.

**Fig. 3:**
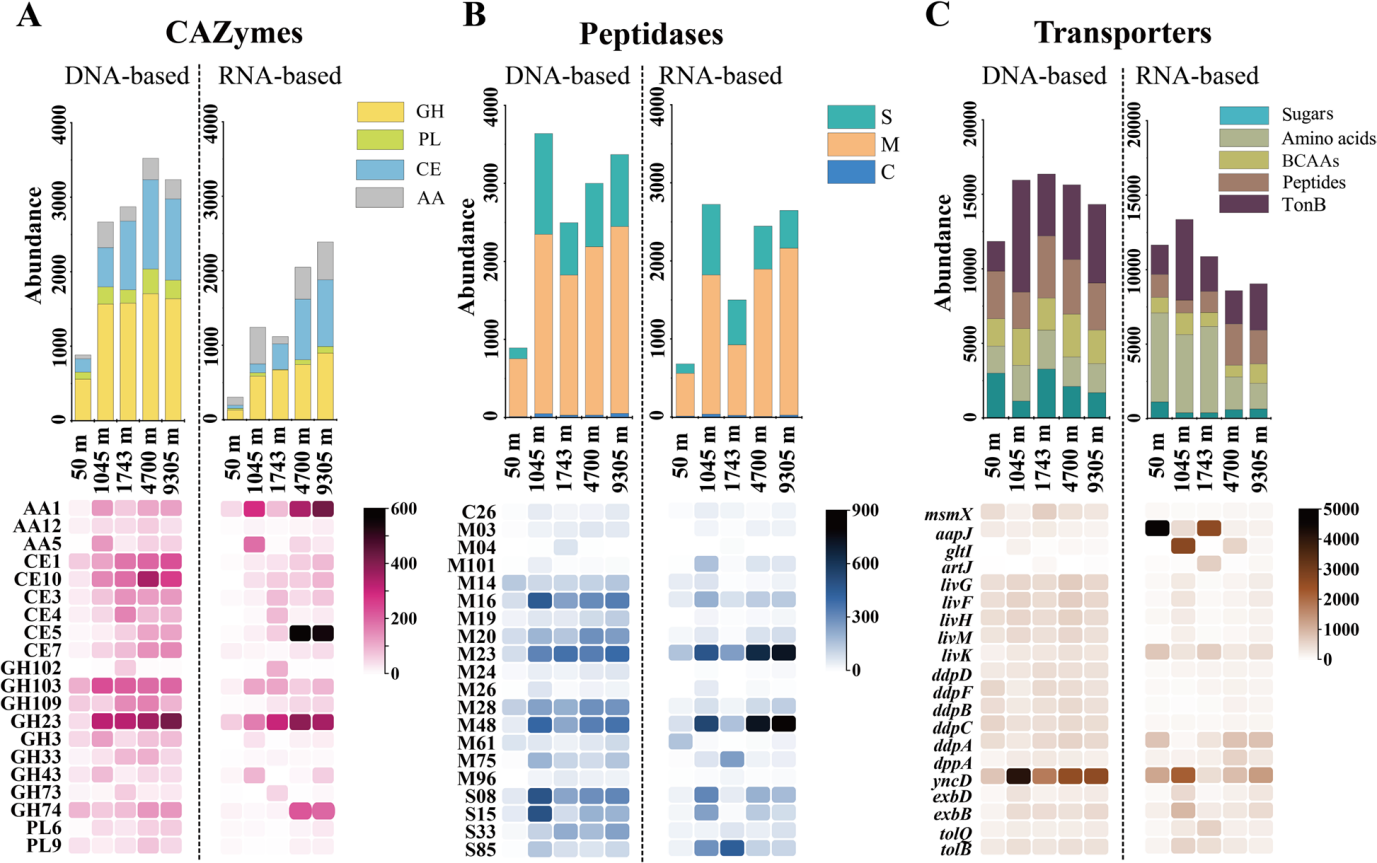
The category and abundance of the genes involved in the polymer degradation and utilization at each depth. **A**,** B**, and **C** The category, DNA-based and RNA-based abundance (represented by TPM value) of the genes that contained/transcribed CAZymes (**A**), peptidases (**B**), and transporters (**C**) genes. **A** CAZyme families: GH: glycoside hydrolase; PL: polysaccharide lyase; CE: carbohydrate esterase; AA: auxiliary activity. **B** Peptidase families: cysteine (C), metallo (M), and serine (S) peptidases. **C** Transporters: sugars (monosaccharides, disaccharides, and oligosaccharides), amino acids, branch-chain amino acids (BCAAs), peptides, and TonB transporters

**Fig. 4:**
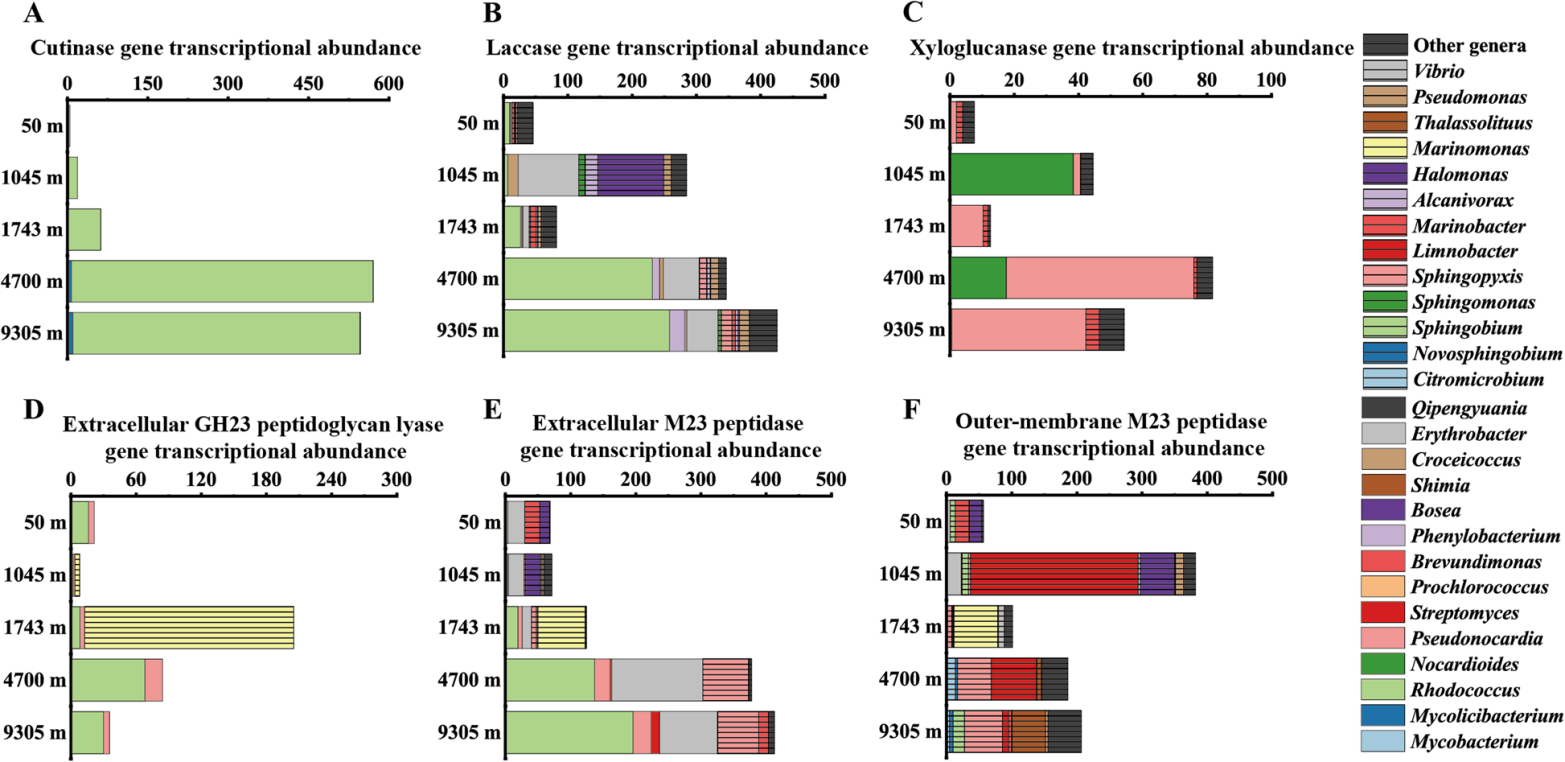
The taxonomic origin and gene transcriptional abundance of cutinase (**A**), laccase (**B**), xyloglucanase (**C**), GH23 peptidoglycan lyase (**D**), extracellular M23peptidase (**E**), and outer-membrane M23 peptidase (**F**) with signal peptides

The total DNA-based abundance of CAZyme genes did not have apparent differences between different depths except at 50 m, while the total RNA-based abundance of CAZyme genes, such as carbohydrate esterase (CE) and glycoside hydrolase (GH) family genes, increased at 4700 m and 9305 m (Fig. 3A and Table S5). In particular, genes encoding cutinase from CE5 (responsible for hydrolyzing ester bonds of cutin, a polymeric structural component of plant cuticle), xyloglucanase from GH74 (responsible for endo-hydrolyzing 1,4-β-D-glucosidic bond in xyloglucan, a hemicellulose polysaccharide in plant cell walls), and laccase from auxiliary activity (AA) 1 (redox enzymes that act in conjunction with CAZymes to degrade organic biopolymers, such as lignin and cellulase) families were particularly active at 4700 m and 9305 m [36–38]. The presence of these highly transcribed genes implied that their target substrates, such as phytoplankton-derived polysaccharides cutin, hemicellulose, and lignin, maybe the main biopolymers available for microbes in the abyssopelagic and hadopelagic zones. The microbes involved in the processing of these polysaccharides primarily belong to Actinobacteria, including genera *Rhodococcus*, *Nocardioides*, and *Pseudonocardia* (Fig. 4A–C). In addition, peptidoglycan lyases from GH23 (responsible for hydrolyzing glycosidic bonds within the glycan of peptidoglycan [39]) were active in all seawater samples (Fig. 3A).

The peptidase genes with high RNA-based abundance mainly belonged to Cysteine (C), Metallo (M), and Serine (S) families (Fig. 3B). The genes encoding M23 peptidase (responsible for cleaving peptide bonds within the peptidoglycan [40]) were especially active at 4700 m and 9305 m. Together with the high transcriptional level of GH23 peptidoglycan lyases in deep waters, this implied that deep-sea microbes possess the capacity to degrade the peptide and glycan components of peptidoglycan for nutrition acquisition.

It is worth noting, however, that some M23 peptidases and GH23 peptidoglycan lyases also play roles in cell wall synthesis and remodeling, rather than being solely involved in nutrition acquisition [41, 42]. The GH23 family peptidoglycan lyases associated with cell wall synthesis are often membrane-bound [41]. Subcellular localization prediction showed that a few peptidoglycan lyases are located in the extracellular space (Figure S4A). These extracellular GH23 enzymes, mainly from the genera *Rhodococcus*, *Pseudonocardia*, and *Marinomonas* (Fig. 4D), are likely used by microbes to break down the glycan component of peptidoglycan for nutrient acquisition. Especially at 1743 m, *Marinomonas* transcribed the majority of the GH23 family peptidoglycan lyases, indicating the significant role of this genus in peptidoglycan degradation. Many previously studied M23 peptidases are also located in the extracellular space [41, 42]. In this study, the identified M23 peptidases were also located in the extracellular space and outer membrane (Figure S4B). The extracellular M23 peptidases are mainly from the genera *Rhodococcus*, *Erythrobacter*, and *Sphingopyxis*, while outer membrane-bound M23 peptidases were mainly in the genera *Sphingopyxis* and *Thalassolituus* in the abyssopelagic and hadopelagic zones (Fig. 4E, F). Although subcellular localization analyses cannot definitely determine the physiological function of M23 peptidases, some marine bacteria are known to secrete extracellular M23 peptidases to degrade the peptidoglycan of other microorganisms for use as a nutrient source [43]. Thus, microbes that transcribe the M23 peptidase genes likely possess the ability to degrade and utilize peptidoglycan. In addition, outer membrane-bound M23 peptidase genes also had high transcriptional levels. Some marine bacteria employ a substrate utilization mechanism where surface-associated enzymes bind and partially degrade biopolymers. These partially degraded substrates are then transported into the periplasm for further degradation, as observed in seawater ecosystems [44]. In this study, outer membrane-bound M23 peptidases may be associated with such substrate utilization behaviors in corresponding strains [45]. Notably, the RNA-based abundance of amino acids transporter genes decreased with depth, while that of peptide transporter genes increased at 4700 m and 9305 m, indicating that microbes in the abyssopelagic and hadopelagic zones have a preference for utilizing peptides over amino acids (Fig. 3C). This is likely because, as surface organic matter sinks, the consumption of soluble organic matter reduces the availability of low molecular weight organic matter (such as amino acids) in the deep sea [2, 46]. Consequently, as previously reported, deep-sea microorganisms may exhibit a greater inclination to utilize peptides [5].

Interestingly, the summed RNA-based abundance of genes encoding M23 peptidases and GH23 peptidoglycan lyases increased with depth (Figure S5). Since peptidoglycan is composed of N-acetylmuramic acids (MurNAc), N-acetylglucosamine (GlcNAc), and D-amino-acids (mainly D-glutamate and D-alanine) [39], the activities of these enzymes would release MurNAc, GlcNAc, and D-amino-acids into the environment. Correspondingly, the genes involved in MurNAc and GlcNAc metabolism were active in the deep sea, suggesting that deep-sea microbes can utilize these compounds (Figure S6A). Genes involved in D-amino-acid metabolism, including those encoding glutamate racemase, alanine racemase, and D-alanine transaminase, were also active in the deep sea (Figure S6B). Previous studies have shown that nearly all N-containing biopolymers in the deep sea exist as amides [47], and peptidoglycan is the main source of these amides. Here in this study, EE genes involved in peptidoglycan degradation had high transcriptional levels in the deep sea. These results suggest that peptidoglycan may represent another significant source of biopolymer nutrition available to deep-sea microbes. The active genes involved in peptidoglycan degradation as well as MurNAc, GlcNAc and D-amino-acids metabolism were primarily associated with genera such as *Rhodococcus*, *Erythrobacter* and *Sphingopyxis* at depths of 4700 m and 9305 m (Fig. 4D–F and Figure S7).

### Microbial EEAs and cell-specific EEAs increased in the hadalpelagic zone

**Fig. 5:**
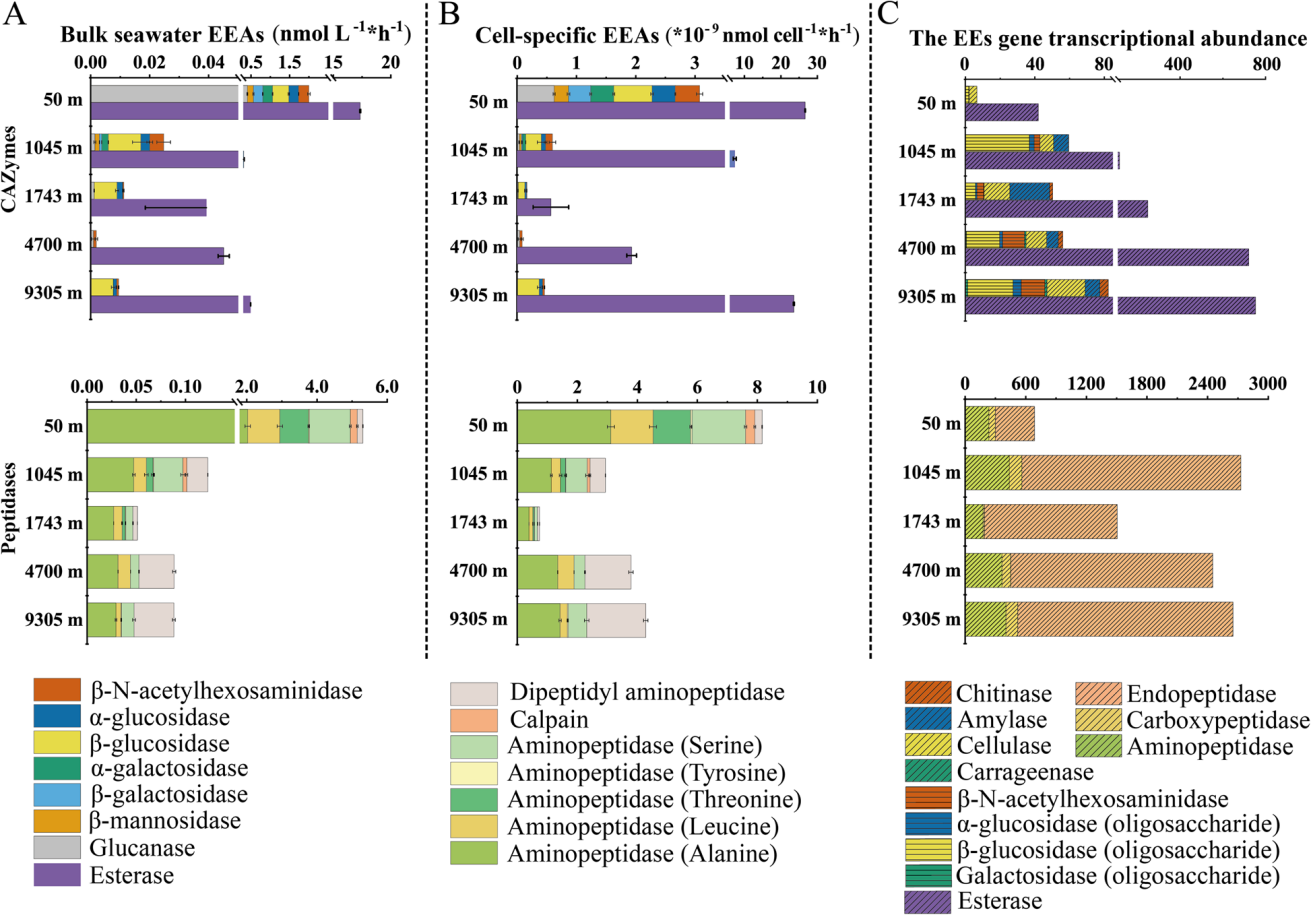
Depth profiles of bulk seawater EEAs (**A**), cell-specific EEAs (**B**), and the transcriptional abundance of corresponding EE genes (**C**)

The analyses described above provide insights into the transcriptional level of EE and transporter genes at different water depths. Next, the EEAs throughout the water column were further investigated (Fig. 5). The hydrolysis rates of 15 fluorogenic substrates were measured to estimate the activities of CAZymes (glycosidase and esterase) and peptidases in seawater (Table S6).

Different categories of EEAs showed different patterns throughout the water column (Fig. 5A). Hydrolysis of all 15 substrates was detected at 50 m and 1045 m while only 11, 7, and 9 substrates were hydrolyzed at 1743 m, 4700 m, and 9305 m, respectively. The highest activity of each EE was observed at 50 m, which may be attributed to the fact that EEAs in the 50 m were measured at in situ temperature (28 ℃) and atmosphere pressure (Table S7), unlike other samples that were tested at in situ high pressure and low temperature (4 ℃). Nevertheless, the second-highest bulk seawater EEAs were observed at 9305 m (0.58 nmol L^−1^*h^−1^). In terms of cell-specific EEAs (the ratio of the substrates’ hydrolysis rates to microbial cell counts), almost all cell-specific EEAs increased in the hadalpelagic zone (9305 m) compared to the abyssopelagic zone (4700 m) (Fig. 5B).

Generally, the changes in bulk seawater EEAs and the cell-specific EEAs were consistent with the changes in the corresponding microbial EE gene transcriptional abundance throughout the water column (Fig. 5C). For example, the total EEAs, the cell-specific EEAs and corresponding EE gene RNA-based abundance of esterase and peptidases increased at 4700 m and 9305 m compared to 1743 m. A previous study found an enhanced proportion of chromophoric dissolved organic matter (CDOM) in the hadal zone of the Mariana Trench [48]. CDOM composition was reported to mainly include aromatic amino acids and lignin phenols [49–52], which likely resulted from the intensified microbial activity in deep waters as detected in this study. Although the EEAs were measured at high pressure (90 MPa) and low temperature (4 ℃) conditions, the summed cell-specific EEAs were the second highest at 9305 m (28.29 *10^−9^ nmol cell^−1^*h^−1^). This was consistent with previous studies, showing that the level of cell-specific EEAs is typically higher in the deep sea than in upper seawater in the Tyrrhenian Sea and the New Britain Trench [53, 54]. This is perhaps not surprising since LMW OM becomes less readily available in the deep sea [46], leading to an increase in the transcription of microbial EE genes to facilitate degradation and nutrition acquisition from more complex biopolymer substrates. The unique funnel-shaped geomorphology of trenches can facilitate the accumulation of POM descending from the surface, potentially including terrestrial OM sources [55]. This gathering process is aided by the lateral movement from the edges and slopes of the trenches, often triggered by seismic activities [56]. In addition, the presence of chemoautotrophic prokaryotes like ammonia-oxidizing archaea, which are known to be abundant in marine water columns, could contribute to the OM pool in the deep sea [14]. Given these factors, it is plausible that OM increases in trenches, potentially leading to the observed elevated EEAs.

## Conclusions

This study represents a comprehensive investigation of the deep-sea microbiome and activities of extracellular enzymes involved in OM processing in the Mariana Trench. The findings highlighted the dominant role of Actinobacteria as the key active microbial population responsible for cycling biopolymers in abyssopelagic and hadopelagic zones. The high transcriptional levels of the genes encoding enzymes such as cutinase, laccase, xyloglucanase, GH23 peptidoglycan lyase, and M23 peptidase suggested that complex phytoplankton polysaccharides and microbial peptidoglycan serve as the primary source of carbon and energy for deep-sea microbes. Additionally, the detection of high cell-specific EEAs as well as the high transcription of microbial EEA gene underscores the microbial capacity to thrive under the challenging conditions of high pressure and low temperature in the hadal zone of the Marina Trench.

## Methods

### Sampling and sample processing

Seawater samples from five depths (50, 1045, 1743, 4700, and 9305 m) were collected using Niskin bottles fitted on a Sea-Bird Carousel equipped with a conductivity-temperature-depth (CTD) sensor (Sea-Bird SBE 911) at a site (11°20′ N, 142°04′ E) of Mariana Trench during the cruise of R/V ‘Hai Da’ from 20 to 21 March 2019. After prefiltration to remove large organisms through a 10-μm-pore-size polycarbonate membrane (142 mm, Millipore Co., USA), 80 L seawater samples were filtered using 0.22-μm-pore-size membranes (142 mm, Millipore Co., USA) to collect the free-living and particle-associated (< 10 μm) microbial cells on board. Two membranes were gathered at each depth and used for metagenomic and metatranscriptomic sequencing, respectively. After filtration, the membranes were quickly frozen using liquid nitrogen and stored at − 80 °C. Then the 80-L filtered seawater samples were concentrated to 80 ml using a tangential flow filtration system with 5000-Dalton hollow modified polyethersulfone membranes (Spectrum Laboratories, Inc., USA) [8]. The concentrated seawater was used for the assays of cell-free EEAs, which represent the majority of total EEAs in oligotrophic seawater and could benefit microbes at the community level [57].

### Bacterial counts by flow cytometry

Subsamples of unconcentrated seawater (1 mL) from each depth were fixed with 0.125% (v/v, final concentration) glutaraldehyde after sampling, and stored at − 80 °C for subsequent analysis using a Flow Cytometry (Merck Millipore, guava easyCyte HT Co., USA). Before counting, samples were stained with SYBR Green (5 × , Invitrogen) for 30 min, and DNA-containing cells were identified based on fluorescence and scatter signals [58].

### Metagenomic sequencing and analyses

Microbial DNA was extracted from membrane samples using the E.Z.N.A.® stool DNA Kit (Omega Bio-tek, Norcross, GA, USA) according to the manufacturer’s protocols. Metagenomic shotgun sequencing libraries were constructed and sequenced at Shanghai Biozeron Biological Technology Co. Ltd. The libraries were sequenced on an Illumina MiSeq platform and 150 bp paired-end reads were generated. Approximately 50 Gbp raw sequencing data were obtained for each sample. Raw sequence reads underwent quality trimming using Trimmomatic (v0.38) to remove adaptor contaminants and low-quality reads [59]. Taxonomic profiling of clean reads for each sample was determined by Kraken [60]. The 16S miTags were extracted from metagenomic clean reads using phyloFlash (v3.4.2) with default parameters and classified with the SILVA v138.1 database [61]. Genera that were common contaminants in the laboratory environment but not representative of marine bacteria, such as *Staphylococcus* and *Bradyrhizobium*, were removed. Clean reads were assembled into contigs using IDBA-UD and contigs greater than 500 bp were retained (v1.1.1) (Table S8) [62]. The total length of assembled contigs of the 50-m sample was relatively small, which should be due to the sequence microdiversity of SAR11 clades and *Prochlorococcus* that are abundant in surface seawater [63–65]. Indeed, the numbers of contigs less than 500 bp were much more at 50 m than at other samples and taxonomic classification showed that contigs less than 500 bp at 50 m were mainly from *Candidatus* Pelagibacter (SAR11 clades) and *Prochlorococcus* (Figure S8). The open reading frames (ORFs) of assembled contigs were predicted using Prodigal with default parameters (v2.6.3) [66]. ORFs of all samples were clustered to generate a non-redundant gene set using CD-HIT with a 0.95 sequence identity threshold (v4.8.1) [67]. The DNA-based gene abundance in each sample was estimated by the number of mapped reads and normalized as follows: TPM = 10^6^ × (mapped reads/gene length)/sum of (mapped reads/gene length). The reads were mapped to the non-redundant gene set with SOAP2 with default parameters [68, 69], and the TPM value of each gene in each sample was calculated. The TPM value was used to compare the change in the abundance of each gene in different samples.

### Metatranscriptomic sequencing and analyses

Total RNA was extracted using the Soil RNA kit (Omega, USA) from membranes, and sent to WHBioacme (Wuhan, China) for metatranscriptomic sequencing. The rRNAs were removed with Ribo-Zero rRNA Removal Kits (Illumina, San Diego, CA, USA). A TruSeq RNA Sample Prep Kit (Illumina, San Diego, CA, USA) was utilized for constructing the RNA sequencing library. The libraries were sequenced on an Illumina HiSeq TM2000 platform and 150 bp paired-end reads were generated. Approximately 50 Gbp raw sequencing data were obtained for each sample. Raw sequencing reads were quality controlled using Trimmomatic (v0.38) to remove poor quality reads, short reads and reads without a counterpart. Then the quality-filtered reads were sorted using SortMeRNA (v2.1) to remove rRNA reads [70]. The RNA-based taxonomic profiling of each sample was conducted using Kraken software based on high-quality non-rRNA clean reads. The RNA-based gene abundance in each sample was estimated by mapping the non-rRNA clean reads to a non-redundant gene set with SOAP2, and TPM for each gene was calculated as described above.

### Functional gene annotation

Taxonomic annotation of the functional gene was determined by Kraken with default parameters (Table S9). The signal peptides were predicted by SignalP (v5.0) and further finer subcellular localization of proteins with signal peptides was predicted using PSORTb with default parameters [71, 72]. Carbohydrate-Active enzymes were identified by searching the Carbohydrate-Active enzymes (CAZy) database with HMMER 3.3 (*E *value < 1e^−10^) [73]. The peptidases were annotated by BLASTP (*E *value < 1e^−10^, identity > 50%) search against the MEROPS database (Release 11.0) [74]. Annotation of transporters was obtained from the KEGG Automatic Annotation Server (KAAS) [75, 76].

### Measurements of enzymatic activities

Fifteen fluorogenic substrates, including eight 4-methylumbelliferyl (MUF) labeled substrates and seven 7-amido-4-methylcoumarin (MCA) labeled substrates, were used to measure the EEAs in different seawater samples (Table S6) [77, 78]. All chemicals were obtained from Sigma-Aldrich (USA).

Enzymatic activities were assayed in the simulated in situ temperature and pressure conditions (Table S7). The high pressure was applied with a water pump in high-pressure vessels (Feiyu Science and Technology Exploitation Co., Ltd., Nantong, China). The hydrolysis rate of the substrate was used to calculate EEA (nmol L^−1^*h^−1^). Pre-experiment was conducted to explore the optimal measurement conditions, mainly the incubation time. Typically, 50 μl of 1 mM fluorogenic substrate and 150 μl of concentrated seawater sample were mixed in a 1-ml syringe and the syringe was then immediately put in the reaction vessel. Seawater sample from 50 m depth was incubated for 6 h at 28 ℃, and others were incubated for 168 h at 4 ℃ [79]. After the reaction, fluorescence was detected using a spectrofluorometer (Hitachi, model F-4500) at 365 nm excitation and 445 nm emission. Autoclaved seawater was used as a negative control. The enzyme activity detection of each sample was repeated three times, each with three parallels. The calibration curves were produced to allow the conversion of fluorescence intensity into product concentration using a series of standard solutions of 4-methylumbelliferone (MUF; Sigma) and 7-amino-4-methylcoumarin (MCA; Sigma) in seawater.

Given the limited availability of samples and previous studies indicating that the majority of the deep-sea EEAs are found in the dissolved fraction of seawater, we chose to use the filtered seawater for EEA measurements [80, 81]. Furthermore, due to the low concentration of EEs in deep-sea environments, the filtered seawater was concentrated to enhance the detection of a wider range of enzymatic activities. This approach not only reduced incubation time but also standardized the measurement conditions for various enzymes. Generally, the EEAs measured in this study fell within the same order of magnitude as those in previous studies [53, 54], supporting that the treatment did not significantly alter the enzyme activities under investigation.

### Supplementary Information

**Supplementary Material 1.**

## Data Availability

Sequence data for metagenome and metatranscriptome have been deposited in the Genome Sequence Archive [82] in National Genomics Data Center [83], China, under accession numbers CRA009089 and CRA009090, respectively.
