# Supplementary material for: Phytoplankton-derived polysaccharides and microbial peptidoglycans are key nutrients for deep-sea microbes in the Mariana Trench
Source: Microbiome. 2024 Apr 25;12:77. doi: 10.1186/s40168-024-01789-x (PMC11044484; doi:10.1186/s40168-024-01789-x)
Supplement: Supplementary file 2 — Supplementary Material 1. [file 40168_2024_1789_MOESM1_ESM.docx]

**Additional file 1:**

**Phytoplankton-derived polysaccharides and microbial peptidoglycans are key nutrients for deep-sea microbes in the Mariana Trench**

Yan-Ru Dang^1^, Qian-Qian Cha^1^, Sha-Sha Liu^1^, Shu-Yan Wang^2^, Ping-Yi Li^1,3^, Chun-Yang Li^2,3^, Peng Wang^2,3^, Xiu-Lan Chen^1,3^, Ji-Wei Tian^2^, Yu Xin^2^, Yin Chen^2,4^*, Yu-Zhong Zhang^2,3,5^*, Qi-Long Qin^1,3^*

^1^State Key Laboratory of Microbial Technology, Shandong University, Qingdao, China.

^2^College of Marine Life Sciences & Frontiers Science Center for Deep Ocean Multispheres and Earth System, Ocean University of China, Qingdao, China

^3^Laboratory for Marine Biology and Biotechnology, National Laboratory for Marine Science and Technology, Qingdao, China.

^4^School of Life Sciences, University of Warwick, Coventry CV4 7AL, United Kingdom.

^5^Marine Biotechnology Research Center, State Key Laboratory of Microbial Technology, Shandong University, Qingdao, China

* Corresponding author: Yin Chen, y.chen.25@warwick.ac.uk; Yu-Zhong Zhang, zhangyz@sdu.edu.cn; Qi-Long Qin, qinqilong@sdu.edu.cn

**Supplementary Figures**

**Fig. S1:**

**
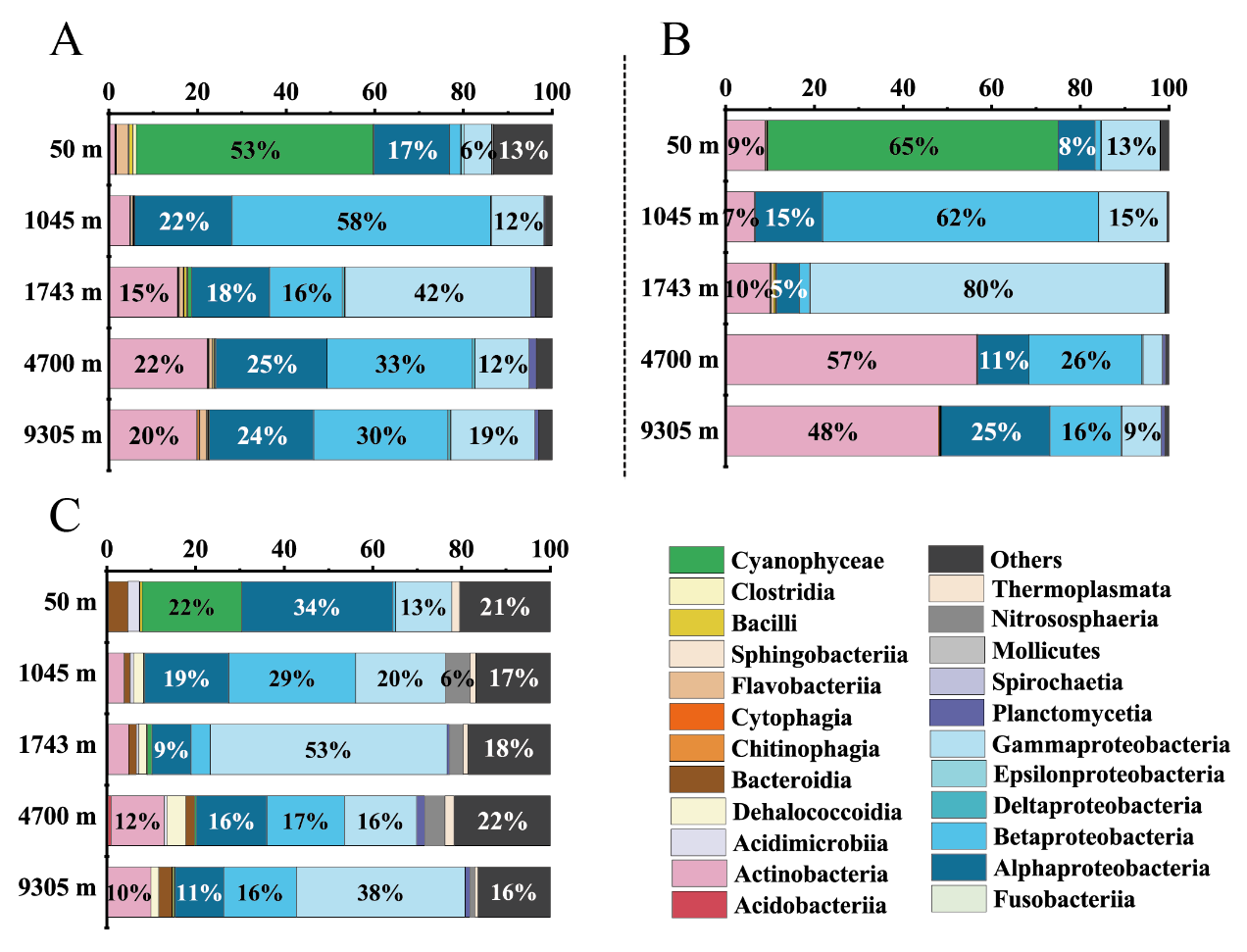
**

**Fig. S1** Abundance profiling of the microbial community at class level based on non-redundant gene set and 16S miTags. **A** The relative abundance of microbial community at class level. **B** The relative transcriptional abundance of microbial community at class level. **C** The relative abundance of microbial community at class level based on 16S miTags.

**Fig. S2:**

**
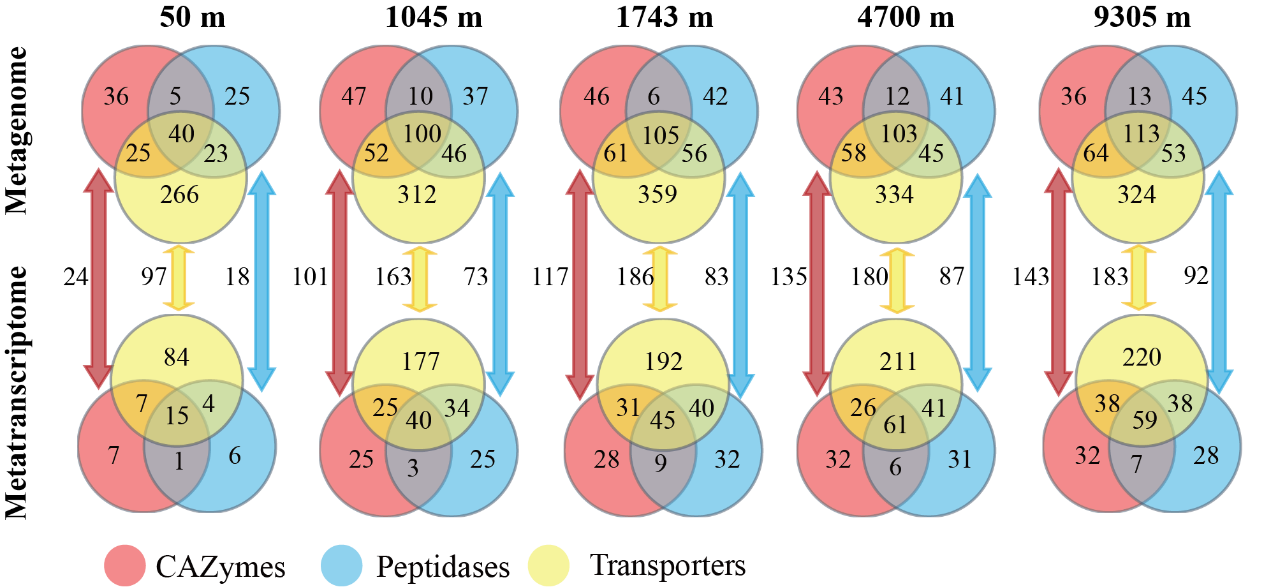
**

**Fig. S2** Venn diagram of the genera that contained (deduced from metagenomic data) or transcribed (deduced from metatranscriptomic data) the genes of CAZymes, peptidases and transporters. Numbers in red, blue and yellow circles represent the numbers of the genera that contained/transcribed CAZymes, peptidases and transporters genes, respectively. Numbers in circle overlaps represent the number of the genera that contained/transcribed two or three types of these genes. Numbers in double-headed arrows represent the number of the genera both containing and transcribing the same kind of genes.

**Fig. S3:**

**
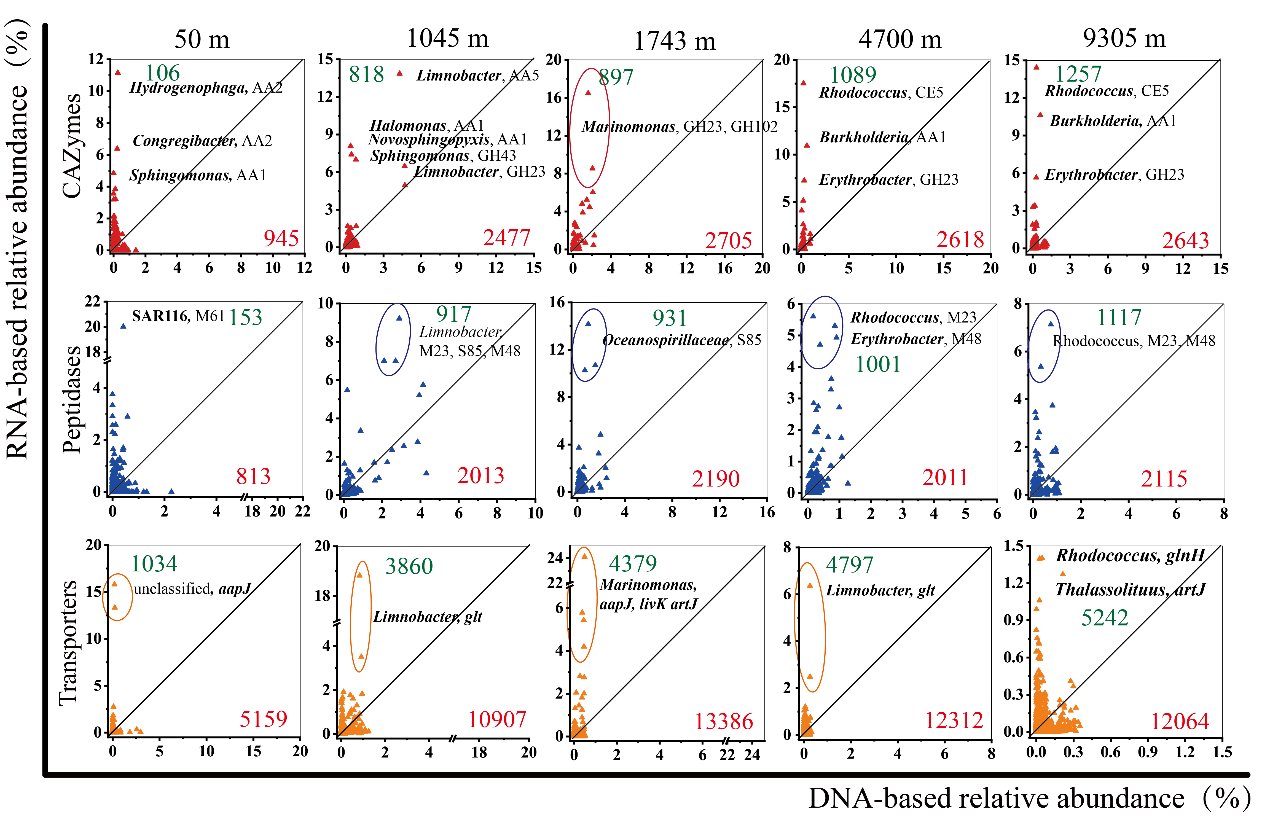
**

**Fig. S3** Correlation between DNA-based and RNA-based abundance of the main active genes involved in polymers degradation and utilization at each depth. The genes in a hollow circle are from the same genus. The red and green digits represent the numbers of encoded and transcribed genes, respectively.

**Fig. S4:**

**
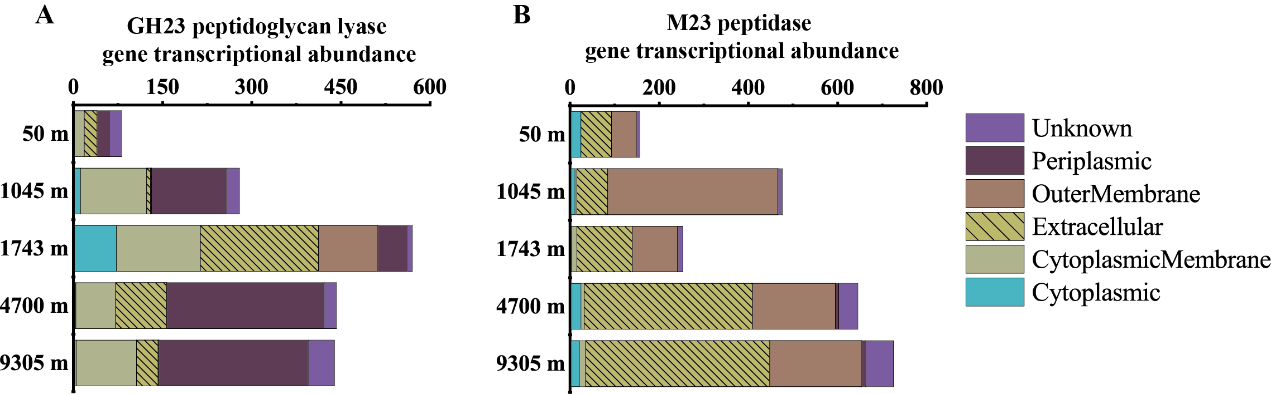
**

**Fig. S4** Subcellular localization prediction and gene transcriptional abundance of GH23 peptidoglycan lyase (**A**) and M23 peptidase (**B**) with signal peptide.

**Fig. S5:**

**
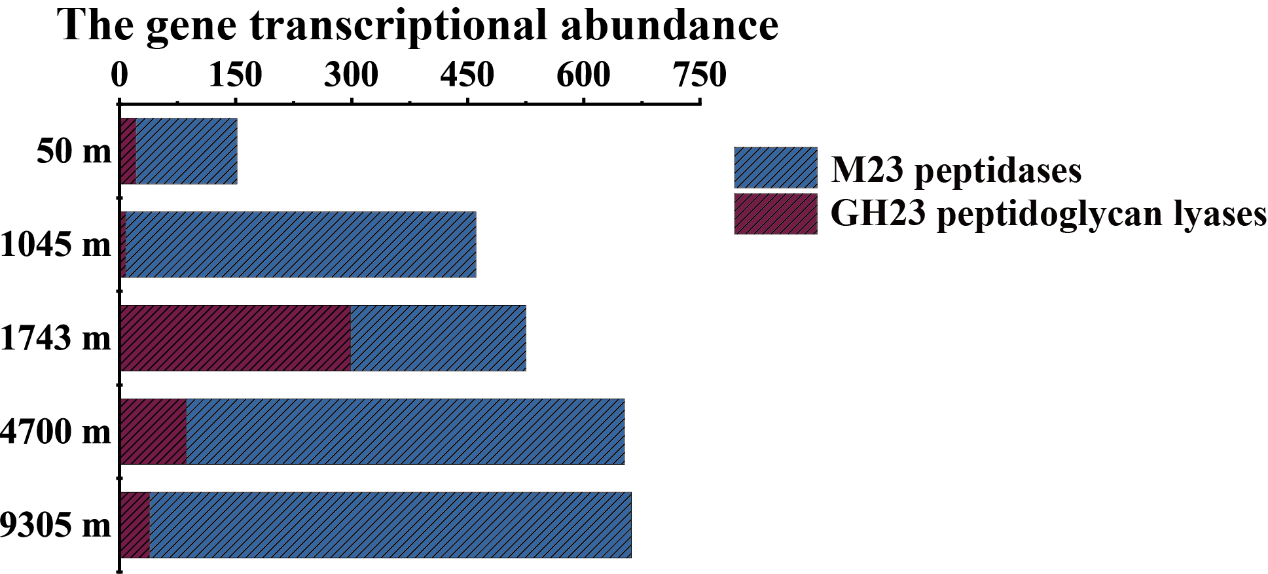
**

**Fig. S5** The summed transcriptional abundance of extracellular and outer-membrane-bound M23 peptidase and extracellular GH23 peptidoglycan lyase genes.

**Fig. S6:**

**
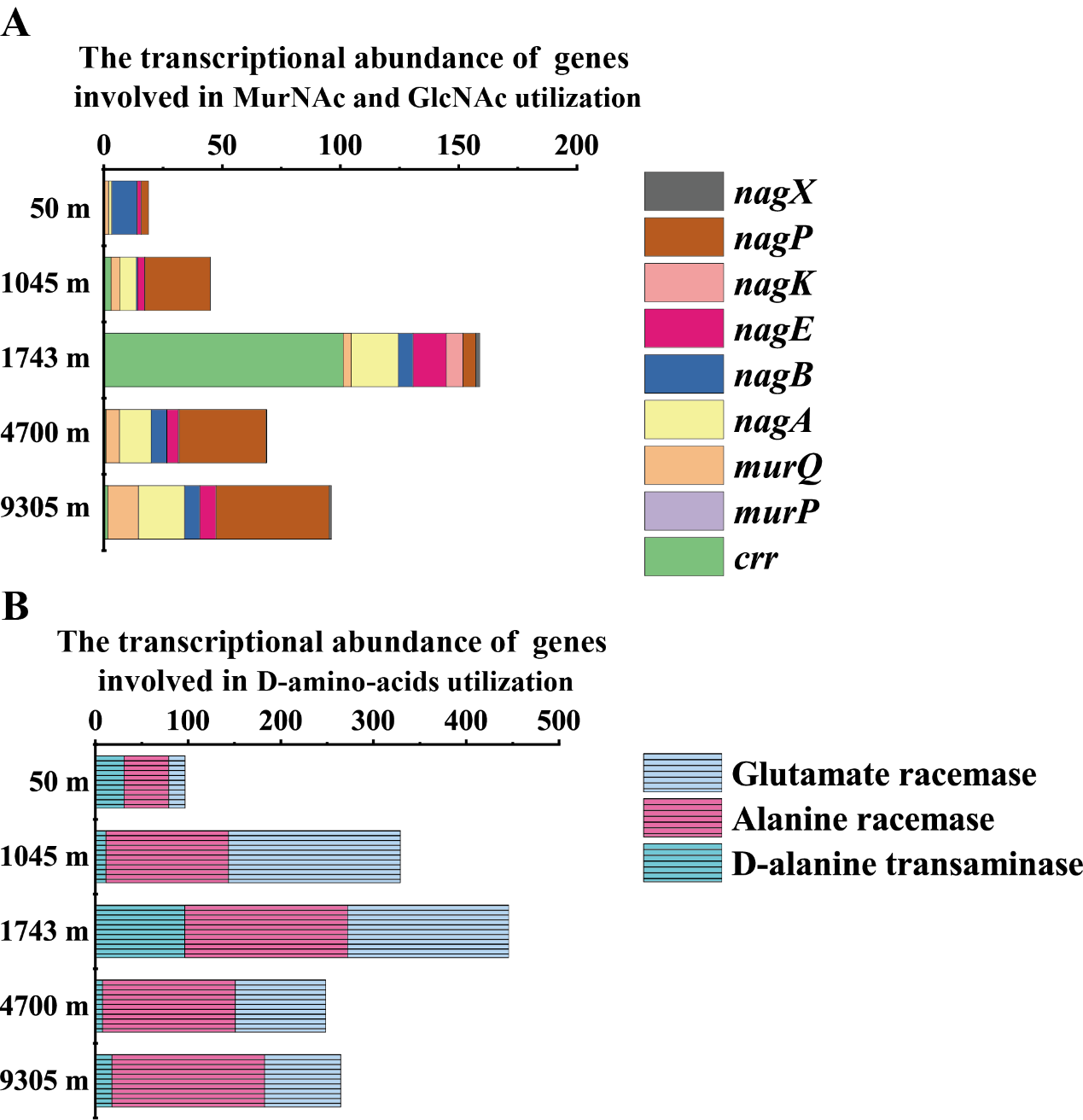
**

**Fig. S6** The transcriptional abundance of genes involved in N-acetylmuramic acid (MurNAc) and N-acetylglucosamine (GlcNAc) utilization (**A**), as well as D-amino-acids utilization (**B**). The gene *ccr* was involved in transport and phosphorylation of MurNAc and GlcNAc during utilization. The *mur* and *nag* genes cluster involved in MurNAc and GlcNAc utilization, respectively.

**Fig. S7:**

**
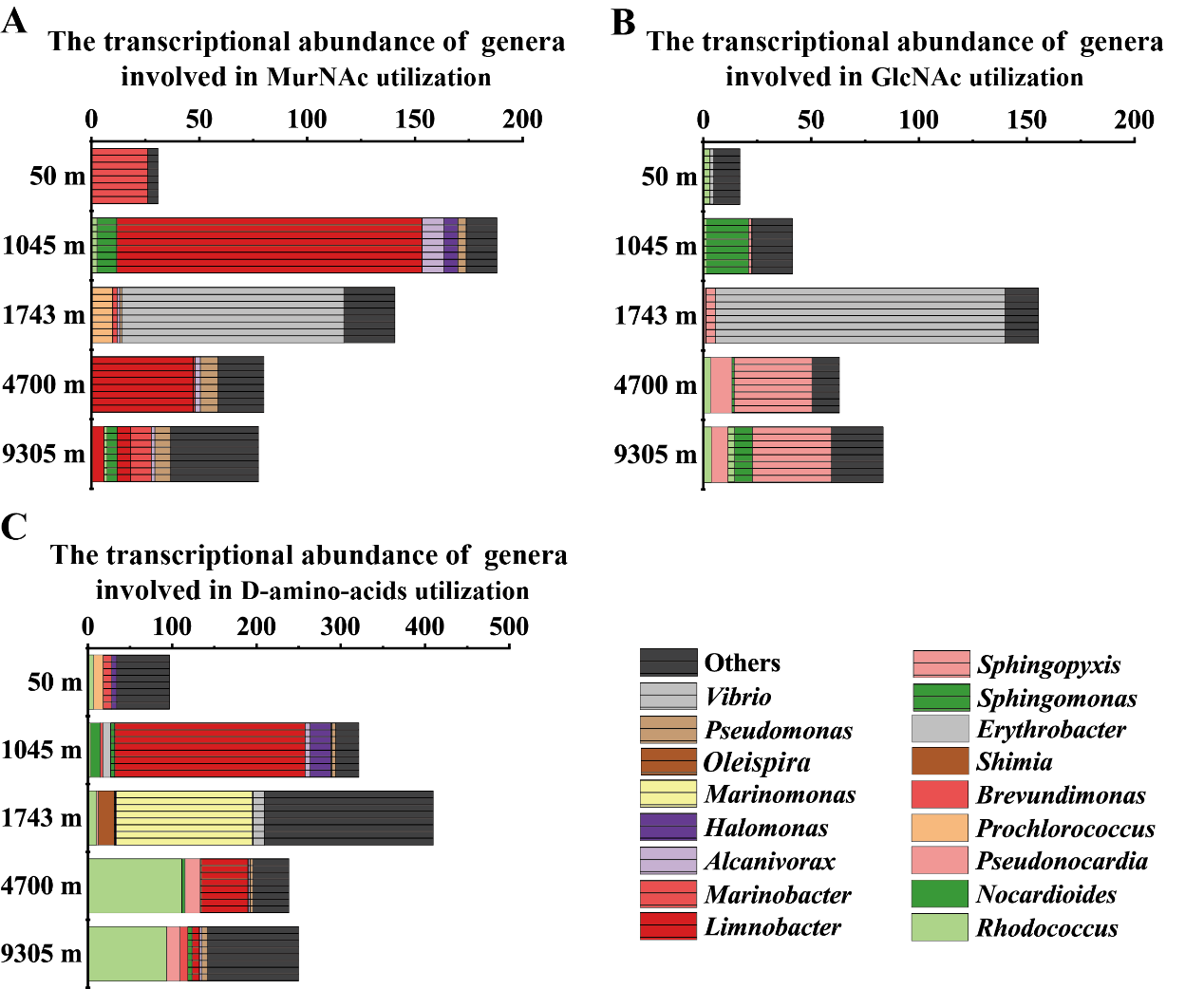
**

**Fig. S7** The taxonomic origin and transcriptional abundance of the genes involved in N-acetylmuramic acid (MurNAc, **A**), N-acetylglucosamine (GlcNAc, **B**) and D-amino-acids (**C**) utilization.

**Fig. S8:**

**
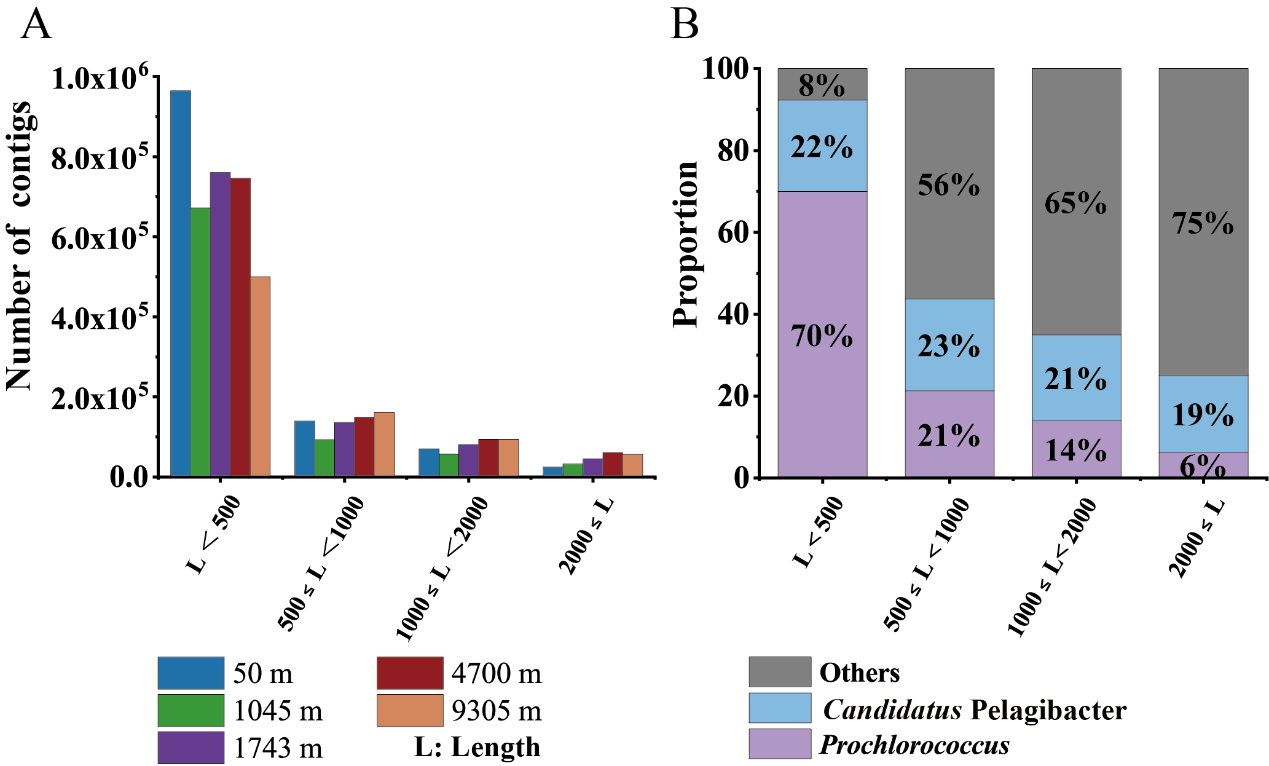
**

**Fig. S8** The number and taxonomic classification of contigs with different length from 50 m sample. **A** The number of contigs with different length from each sample. **B** The taxonomic classification of different length contigs from 50 m sample at Genus level.

**Supplementary Tables**

**Table S1** Taxonomic classification and mapping information of metagenomic sequencing reads**.**

| Depth (m) | Number of total reads | Taxonomic classification (%) | Genus-level classification (%) | Reads assembled into contigs (%) | Reads mapped to gene catalogs (%) |
| --- | --- | --- | --- | --- | --- |
| 50 | 103,239,308 | 30.74 | 24.22 | 10.99 | 8.37 |
| 1045 | 92,068,759 | 47.94 | 43.13 | 38.49 | 29.07 |
| 1743 | 120,417,527 | 27.42 | 21.84 | 37.30 | 28.22 |
| 4700 | 106,851,470 | 30.80 | 26.33 | 37.30 | 28.67 |
| 9305 | 97,668,837 | 35.51 | 32.36 | 41.14 | 31.64 |

**Table S2** Taxonomic classification and mapping information of metatranscriptomic sequencing reads**.**

| Depth (m) | Number of clean reads | Number of non-rRNA reads | Taxonomic classification (%) | Genus-level classification (%) | Reads mapped to contigs (%) | Reads mapped to gene catalogs (%) |
| --- | --- | --- | --- | --- | --- | --- |
| 50 | 116,908,235 | 41,039,883 | 6.42 | 6.02 | 2.16 | 0.85 |
| 1045 | 120,950,687 | 95,609,337 | 78.60 | 77.17 | 67.68 | 51.60 |
| 1743 | 133,100,926 | 87,933,722 | 49.01 | 46.71 | 64.69 | 44.83 |
| 4700 | 119,248,295 | 46,721,324 | 53.88 | 51.18 | 40.93 | 26.58 |
| 9305 | 127,802,312 | 54,551,812 | 57.68 | 54.60 | 37.51 | 22.73 |

**Table S3** Contribution of the sequencing reads of the top five genera to the taxonomic sequencing reads and total sequencing reads in metagenomic data.

|  | % of taxonomic sequencing reads | | | | |  | % of total sequencing reads | | | | |
| --- | --- | --- | --- | --- | --- | --- | --- | --- | --- | --- | --- |
|  | 50 m | 1045 m | 1743 m | 4700 m | 9305 m |  | 50 m | 1045 m | 1743 m | 4700 m | 9305 m |
| *Rhodococcus* | 2.26 | 0.65 | **3.95** | **21.41** | **16.56** |  | 0.15 | 0.51 | **1.94** | **11.53** | **9.55** |
| *Nocardioides* | 0.14 | **3.74** | 0.06 | **1.59** | 0.16 |  | 0.01 | **2.94** | 0.03 | **0.86** | 0.09 |
| *Pseudonocardia* | 0.65 | 0.70 | **5.41** | **24.46** | **14.54** |  | 0.04 | 0.55 | **2.65** | **13.18** | **8.39** |
| *Prochlorococcus* | **38.95** | 0.05 | 0.71 | 0.35 | 0.07 |  | **2.50** | 0.04 | 0.35 | 0.19 | 0.04 |
| *Brevundimonas* | 0.57 | 1.10 | 0.05 | 0.19 | **5.77** |  | 0.04 | 0.86 | 0.02 | 0.10 | **3.33** |
| *Methylobacterium* | 0.20 | 0.35 | 0.02 | 0.15 | **8.03** |  | 0.01 | 0.27 | 0.01 | 0.08 | **4.63** |
| *Erythrobacter* | 0.55 | **4.67** | 0.23 | 1.20 | 0.76 |  | 0.04 | **3.67** | 0.11 | 0.64 | 0.44 |
| *Sphingomonas* | 0.38 | **6.08** | 0.10 | 0.38 | 3.28 |  | 0.02 | **4.78** | 0.05 | 0.20 | 1.89 |
| *Sphingopyxis* | 0.09 | 0.35 | 0.63 | **3.08** | **4.90** |  | 0.01 | 0.27 | 0.31 | **1.66** | **2.82** |
| *Limnobacter* | 0.01 | **57.02** | 0.80 | **17.47** | 4.09 |  | 0.00 | **44.82** | 0.39 | **9.41** | 2.36 |
| *Alteromonas* | **11.56** | 0.05 | 0.72 | 0.01 | 0.02 |  | **0.74** | 0.04 | 0.35 | 0.01 | 0.01 |
| *Marinobacter* | **3.92** | 0.20 | 2.13 | 0.85 | 1.63 |  | **0.25** | 0.15 | 1.04 | 0.46 | 0.94 |
| *Pseudoalteromonas* | **2.87** | 0.04 | 2.11 | 0.07 | 0.04 |  | **0.18** | 0.03 | 1.04 | 0.04 | 0.02 |
| *Halomonas* | **3.10** | **9.19** | 0.75 | 0.49 | 0.29 |  | **0.20** | **7.22** | 0.37 | 0.26 | 0.17 |
| *Marinomonas* | 0.10 | 0.43 | **28.76** | 0.01 | 0.04 |  | 0.01 | 0.34 | **14.10** | 0.01 | 0.02 |
| *Oleispira* | 0.00 | 0.02 | **5.48** | 0.05 | 0.05 |  | 0.00 | 0.01 | **2.68** | 0.03 | 0.03 |
| *Vibrio* | 0.15 | 0.14 | **15.45** | 0.03 | 0.06 |  | 0.01 | 0.11 | **7.57** | 0.02 | 0.04 |

**Note:** The genera abundances of the top five genera in each sample are shown in bold.

**Table S4** Contribution of the sequencing reads of the top five genera to the taxonomic sequencing reads and non-rRNA sequencing reads in metatranscriptomic data.

|  | % of taxonomic sequencing reads | | | | |  | % of non-rRNA sequencing reads | | | | |
| --- | --- | --- | --- | --- | --- | --- | --- | --- | --- | --- | --- |
|  | 50 m | 1045 m | 1743 m | 4700 m | 9305 m |  | 50 m | 1045 m | 1743 m | 4700 m | 9305 m |
| *Rhodococcus* | 2.26 | 0.65 | **3.95** | **21.41** | **16.56** |  | 0.15 | 0.51 | **1.94** | **11.53** | **9.55** |
| *Nocardioides* | 0.14 | **3.74** | 0.06 | **1.59** | 0.16 |  | 0.01 | **2.94** | 0.03 | **0.86** | 0.09 |
| *Pseudonocardia* | 0.65 | 0.70 | **5.41** | **24.46** | **14.54** |  | 0.04 | 0.55 | **2.65** | **13.18** | **8.39** |
| *Prochlorococcus* | **38.95** | 0.05 | 0.71 | 0.35 | 0.07 |  | **2.50** | 0.04 | 0.35 | 0.19 | 0.04 |
| *Brevundimonas* | 0.57 | 1.10 | 0.05 | 0.19 | **5.77** |  | 0.04 | 0.86 | 0.02 | 0.10 | **3.33** |
| *Methylobacterium* | 0.20 | 0.35 | 0.02 | 0.15 | **8.03** |  | 0.01 | 0.27 | 0.01 | 0.08 | **4.63** |
| *Erythrobacter* | 0.55 | **4.67** | 0.23 | 1.20 | 0.76 |  | 0.04 | **3.67** | 0.11 | 0.64 | 0.44 |
| *Sphingomonas* | 0.38 | **6.08** | 0.10 | 0.38 | 3.28 |  | 0.02 | **4.78** | 0.05 | 0.20 | 1.89 |
| *Sphingopyxis* | 0.09 | 0.35 | 0.63 | **3.08** | **4.90** |  | 0.01 | 0.27 | 0.31 | **1.66** | **2.82** |
| *Limnobacter* | 0.01 | **57.02** | 0.80 | **17.47** | 4.09 |  | 0.00 | **44.82** | 0.39 | **9.41** | 2.36 |
| *Alteromonas* | **11.56** | 0.05 | 0.72 | 0.01 | 0.02 |  | **0.74** | 0.04 | 0.35 | 0.01 | 0.01 |
| *Marinobacter* | **3.92** | 0.20 | 2.13 | 0.85 | 1.63 |  | **0.25** | 0.15 | 1.04 | 0.46 | 0.94 |
| *Pseudoalteromonas* | **2.87** | 0.04 | 2.11 | 0.07 | 0.04 |  | **0.18** | 0.03 | 1.04 | 0.04 | 0.02 |
| *Halomonas* | **3.10** | **9.19** | 0.75 | 0.49 | 0.29 |  | **0.20** | **7.22** | 0.37 | 0.26 | 0.17 |
| *Marinomonas* | 0.10 | 0.43 | **28.76** | 0.01 | 0.04 |  | 0.01 | 0.34 | **14.10** | 0.01 | 0.02 |
| *Oleispira* | 0.00 | 0.02 | **5.48** | 0.05 | 0.05 |  | 0.00 | 0.01 | **2.68** | 0.03 | 0.03 |
| *Vibrio* | 0.15 | 0.14 | **15.45** | 0.03 | 0.06 |  | 0.01 | 0.11 | **7.57** | 0.02 | 0.04 |

**Note:** The genera transcriptional abundances of the top five genera in each sample are shown in bold.

**Table S5** The percent changes of DNA-based and RNA-based abundance of various genes between depths.

|  |  | Metagenome | | | | |  | Metatranscriptome | | | | |
| --- | --- | --- | --- | --- | --- | --- | --- | --- | --- | --- | --- | --- |
|  |  | 50 m | 1045 m | 1743 m | 4700 m | 9305 m |  | 50 m | 1045 m | 1743 m | 4700 m | 9305 m |
| CAZymes | GH | 77% | 64% | 68% | 60% | 63% |  | 63% | 56% | 71% | 45% | 46% |
|  | PL | 8% | 7% | 7% | 10% | 9% |  | 6% | 3% | 1% | 3% | 4% |
|  | CE | 10% | 18% | 19% | 22% | 21% |  | 12% | 8% | 20% | 32% | 30% |
|  | AA | 5% | 11% | 6% | 8% | 8% |  | 19% | 33% | 8% | 19% | 20% |
| peptidases | C | 1% | 1% | 1% | 1% | 2% |  | 3% | 2% | 2% | 1% | 1% |
|  | M | 83% | 63% | 72% | 72% | 71% |  | 80% | 65% | 60% | 77% | 81% |
|  | S | 16% | 36% | 27% | 27% | 27% |  | 18% | 33% | 38% | 22% | 18% |
| Transporters | Sugars | 25% | 7% | 20% | 14% | 12% |  | 9% | 3% | 3% | 7% | 7% |
|  | Amino acids | 15% | 15% | 16% | 13% | 14% |  | 51% | 39% | 53% | 26% | 19% |
|  | BCAAs | 16% | 15% | 13% | 18% | 16% |  | 9% | 11% | 9% | 9% | 15% |
|  | Peptides | 27% | 15% | 25% | 23% | 22% |  | 13% | 6% | 13% | 32% | 25% |
|  | TonB | 17% | 47% | 25% | 32% | 37% |  | 17% | 41% | 22% | 26% | 35% |

**Note:** GH: glycoside hydrolase; PL: polysaccharide lyase; CE: carbohydrate esterase; AA: auxiliary activity; C: cysteine peptidase; M: metallo peptidase; S: serine peptidase; BCAAs: branch-chain amino acids.

**Table S6** List of fluorogenic substrates used in this study.

| **Fluorogenic substrates** | **Corresponding enzymes** |
| --- | --- |
| MUF-butyrate | Esterase/lipase |
| MUF-α-D-galactopyranoside | α-galactosidase |
| MUF-β-D-galactopyranoside | β-galactosidase |
| MUF-α-D-glucopyranoside | α-glucosidase |
| MUF-β-D-glucopyranoside | β-glucosidase |
| MUF-N-acetyl-β-D-glucosaminide | β-N-acetylhexosaminidase |
| MUF-β-D-mannopyranoside | β-mannosidase |
| MUF-β-D-cellobioside | Glucanase |
| L-Alanine-MCA | Aminopeptidase |
| L-Leucine-MCA | Aminopeptidase |
| L-Serine-MCA | Aminopeptidase |
| L-Threonine-MCA | Aminopeptidase |
| L-Tyrosine-MCA | Aminopeptidase |
| L-Lysine-Alanine-MCA | Dipeptidyl aminopeptidase |
| L-N-Succinyl-Leucine-Tyrosine-MCA | Calpain |
|  |  |

| Depth (m) | *In situ* temperature  (℃) | *In situ* Pressure (MPa) | Detection temperature (℃) | Detection Pressure (MPa) | Bacterial Numbers  (cells ml^−1^) | Bulk seawater  EEAs  (nmol L^-1^*h^-1^) | Cell-specific  EEAs  (10^-9^nmol cell^-1^*h^-1^) |
| --- | --- | --- | --- | --- | --- | --- | --- |
| 50 | 28 | 0.5 | 28 | 0.1 | 650,400 | 24.63 | 37.88 |
| 1045 | 4 | 10 | 4 | 10 | 41,799 | 0.45 | 10.83 |
| 1743 | 3 | 17 | 4 | 17 | 68,969 | 0.10 | 1.47 |
| 4700 | 3 | 47 | 4 | 47 | 23,370 | 0.14 | 5.80 |
| 9305 | 2 | 93 | 4 | 90 | 20,617 | 0.58 | 28.29 |

**Table S7** Depth proﬁles of EEA detection parameters, the bacterial cell numbers, bulk seawater EEAs, and cell-specific EEAs of seawater samples in the water column of the Mariana Trench.

**Table S8** General information of the metagenomic data.

| Sample | Clean data (Gb) | Number of contigs (> 500 bp) | Assembly length (bp) | The longest contig (bp) | N50 (bp) | Average G+C contents (%) |
| --- | --- | --- | --- | --- | --- | --- |
| 50 m | 43.42 | 234,717 | 289,218,462 | 134,350 | 1,288 | 37.10 |
| 1045 m | 38.10 | 183,715 | 313,284,711 | 384,164 | 2,159 | 56.60 |
| 1743 m | 46.57 | 202,034 | 447,971,208 | 957,770 | 2,166 | 55.10 |
| 4700 m | 47.60 | 304,865 | 569,255,137 | 677,411 | 2,581 | 58.00 |
| 9305 m | 46.72 | 312,332 | 556,001,574 | 927,100 | 2,367 | 59.00 |

**Table S9** Taxonomic classification percentages of unigenes, CAZyme, peptidase and transporter genes**.**

|  | Number of total genes | Taxonomic classification (%) | Genus-level classification (%) |
| --- | --- | --- | --- |
| Unigenes | 1,715,341 | 26.45 | 23.65 |
| CAZyme genes | 3,505 | 37.20 | 34.78 |
| Peptidase genes | 3,059 | 49.43 | 47.17 |
| Transporter genes | 19,099 | 59.30 | 53.92 |
